# What Are the Needs of City Dwellers in Terms of the Development of Public Spaces? A Case Study of Participatory Budgeting in Częstochowa, Poland

**DOI:** 10.3390/ijerph19095171

**Published:** 2022-04-24

**Authors:** Katarzyna Kołat, Marek Furmankiewicz, Magdalena Kalisiak-Mędelska

**Affiliations:** 1 Institute of Spatial Management, Wroclaw University of Environmental and Life Sciences, Grunwaldzka St. 55, 50-357 Wroclaw, Poland; kolatkatarzyna@gmail.com; 2 Department of Applied Economics, Wroclaw University of Environmental and Life Sciences, Grunwaldzki Sq. 24A, 50-363 Wroclaw, Poland; magdalena.kalisiak-medelska@upwr.edu.pl

**Keywords:** public spaces management, participatory budgeting, community needs, demographic structures, urban districts development, Częstochowa, Poland

## Abstract

Participatory Budgeting (PB) is considered a human-centered method of public resource management and investment planning, which strongly reflects the needs of the inhabitants of the municipality. The aim of this article is to assess the structure of the inhabitants’ needs expressed in the PB procedures in Częstochowa, Poland and their relation to the social and demographic characteristics of the city districts. The standard methods of quantitative and qualitative analysis were used (Pearson correlation coefficient and content analysis of the municipal documents), based on the data about: (1) the projects implemented in Częstochowa PB in the years 2015–2019; (2) the age structures and population density in the districts; and (3) qualitative data on district development characteristics. Based on the authors’ typology of projects, it was found that the most popular tasks were related to the comfort and safety of mobility and recreational facilities used for spending free time in public spaces. A relatively lower level of activity of the citizens was found when expressing their needs in central, densely populated districts with a high share of people aged over 65, and a relatively higher level of activity was found in the districts with a high proportion of people aged 0–18 and with lower population density. In the densely populated central districts, relatively high interest in the development of green areas was observed, while in the less populated developing peripheral districts, the preferred infrastructure was related to mobility. These correlations can be logically explained by the conditions related to the development processes of individual districts. The authors conclude that PB can be an important mechanism in determining local needs for the development of public spaces; however, it rewards the needs of the most active social groups.

## 1. Introduction

In the last few decades, interest in participatory methods of local spatial planning and sustainable resources management has grown significantly worldwide [1,2]. This is related to the abandoning of the priority of economic growth measured primarily by the volume of production, income, and other economic and business indicators, for the priority of quality-of-life improvement, the measure of which is meeting local social needs [3]. As a result, a human-centered approach, related more to humanistic values, is developed [4,5]. In this approach, government and public administration are encouraged to meet community needs by facilitating the collaborative definition of local demands, co-creating solutions, and making changes in response to local consultations, voting procedures, or cross-sectoral negotiations [6,7]. Creating participatory budgeting (PB) is considered one of the many possible methods aimed at developing a human-centered approach [8,9], based on which it is the local inhabitants who prepare and choose projects to be implemented by public administration, rather than officials or managers. PB increases the sensitivity of urban policy to local social needs [10].

The general trend of seeking methods to increase the effectiveness and transparency of local governments in meeting the local needs of inhabitants was of great importance for the development of PB [11,12,13]. The efforts were made to introduce administrative procedures in which the inhabitants decide on the local public investments and land development methods and in which the democratic authorities support and implement these arrangements on a yearly basis [14,15,16]. PB allows local communities to report investment needs regarding public resources, participate in the preparation of projects, and then vote on which tasks are to be implemented. The projects that received the most votes are financed by the municipality within the nearest, usually annual, budget [17].

The PB method has been developing since the 1990s in Brazil [18,19]; it has quickly spread to many cities all over the world, especially in Latin America [20] and in Western Europe [21,22]. A little later, this method began to be implemented in the post-socialist countries of Central and Eastern Europe [23,24,25,26] and also on other continents [27,28,29,30]. The diffusion of PB was facilitated by the existing information exchange within the framework of international cooperation between the municipal governments and communities [31,32] and as a result of PB promotion by various international organizations [30]. Many authors indicate that increasing the involvement of inhabitants in local development processes often favors the implementation of the sustainable development idea to a greater extent than the more pro-investment-oriented activities of public authorities or the profit-oriented private sector [33,34,35,36].

In Poland, the first initiatives imitating PB were announced in the years 2010–2012, and in the next few years it became one of the most popular participation initiatives in the process of managing public spaces in municipalities [37,38,39]. In 2020, PB was implemented in all 66 cities with the highest administrative status of poviat (county), including 18 regional capital cities [40,41], and in many other smaller towns [9].

The literature on PB implemented in various cities of the world is extremely abundant. Most authors, however, focus on the socio-political issues related to organizing the process of inhabitants’ participation in the selection of local projects for implementation and its role in local co-management and citizens’ well-being [19,42,43]. The models of social participation and political processes for involving inhabitants in managing local resources are discussed in detail [44,45,46]. Organizational procedures and the activity of residents or all inhabitants in voting are analyzed [23,47,48], along with the financial issues [40,49]. There is much less research on the geographical distribution of local projects in city districts [50]. The source literature also lacks studies attempting to establish correlations between the socio-demographic structures of districts inhabitants and the needs of public space development they express. There are also few analyses of spatial activities carried out by inhabitants in expressing these needs, e.g., assessing whether the inhabitants of densely populated central multi-family housing areas or the less populated peripheral areas remain more active.

In the paper, the authors try to fill in this research gap, focusing on the issues of type structure and spatial distribution of investment projects (the so-called hard projects) selected in the districts characterized by diverse socio-demographic structures, as part of PB procedures in Częstochowa, in the years 2015–2019. The analyzed projects address the development of municipal areas and facilities managed by the municipal authorities (public spaces). The research case study is a medium-sized industrial and service city located in southern Poland. This is an example of a city where public authorities have been interested in increasing community participation in municipal resource management and investment planning for the last 20 years [48,51]. The main research objectives (RO) of this article include:

RO 1: The assessment of correlations between the demographic structures of district inhabitants and their activity in expressing their needs regarding the management of public spaces using the PB method (Section 4.1);

RO 2: The inventory, typology, and analysis of the structure of project types planned and implemented in the city case study, which reflect the priority needs of inhabitants involved in PB processes (Section 4.2);

RO 3: The assessment of correlations between the demographic structures of district inhabitants and the type of selected projects (Section 4.3).

The main research hypothesis of the article is the statement that the local needs presented by the city inhabitants in terms of the development of public spaces, revealed based on the PB procedures, are related, i.e., to the socio-demographic structures of the districts. The study also discusses the possible impact of other local factors on the types of local projects selected for implementation, including the condition of land and infrastructure development as well as legal and administrative restrictions. Getting to know the main needs of the inhabitants and their activity in expressing them, as well as understanding the relationship between the demographic structures of the inhabitants of districts and their needs, may be helpful in formulating urban development strategies and plans.

After reviewing the literature (Section 2), the authors present the applied research qualitative and quantitative methodologies (Section 3) and results (Section 4). In Section 5 (Discussion), the research findings are discussed in relation to the studies conducted by other authors. On this basis, in Section 6 (Conclusions), the postulates regarding the possibility of further in-depth research of the tasks carried out within PB are formulated.

## 2. Literature Review—What Are the Needs of the Inhabitants in Different Cities, Expressed within the Participatory Budgeting?

An increasing number of studies on PB appear in the literature, in which the types of selected projects are analyzed as well as the elements of geographical analysis regarding the activity of voting inhabitants and the distribution of projects in the individual city districts are addressed. The descriptive studies dominate, which analyze, to a small extent, the relationships between the results of PB and the socio-demographic structures of the population or the features of land and infrastructure development. The analyses presented in the world literature most often address ecological projects related to environmental protection and climate change adaptation and mitigation. For example, Gheorghina and Tap [52] analyzed the support for the ecology-oriented projects in participatory budgeting in Cluj-Napoca (Romania) between 2017 and 2019. They found general interest in environmental issues. Falanga et al. [53] analyzed data on local projects in the thematic area “environment, green structure, and energy” from 2008 to 2018 in Lisbon (Portugal) and focused on four items: citizens’ proposals; votes; projects; and public funding. About 38% of citizen proposals were submitted in the analyzed thematic area, while around 27% of the projects were eventually funded; however, the share of this type of project in the budget showed a decreasing tendency. Most of these types of projects concerned the improvement or creation of new parks and gardens, and the regeneration and green recovery of streets, squares, and public spaces. Cabannes [29] analyzed the extent to which PB contributed to climate change adaptation and mitigation, based on the analysis of 4400 projects from fifteen cities and regions (inhabited by between 26,000 and four million people) in the global South and North (including five cases from Europe). He found that PB initiatives related to climate change did not emerge in response to international priorities but as a citizen and local government reaction to very precise and immediate local climatic effects, so the local needs were the most important.

In Polish literature, there is particularly great interest in PB as it is a relatively new form of increasing social participation in this country. Many works present case studies of individual cities or comparative studies that analyze the structures of the implemented project types. However, there are much fewer analyses of the reasons for such differences, whether they are related only to the condition of public infrastructure in a given city or if the differences also result from various socio-demographic structures of the inhabitants as well as the existing cultural conditions.

In the majority of the analyzed cities, the dominant type of selected projects has been road infrastructure, related to the mobility of inhabitants. For example, Polko [54] observed in Dąbrowa Górnicza (118,285 inhabitants in 2020) that the most frequently selected projects were “roads, parking lots, and pavements” (33.8% of accepted projects) and “leisure time (play-grounds, gyms)” (27.7%). Similarly, in Katowice PB (290,553 inhabitants in 2020), in the years 2015–2017, most of the projects concerned the renovation of neglected road infrastructure, pavements, and pedestrian routes [55]. Similarly, in Wrocław (641,928 inhabitants in 2020), in the PB 2014, most of the funds were invested in roads (28%), followed by recreational facilities (19%) and educational facilities (connected with schools) with their neighboring infrastructure (15%) [50,56,57].

Wójcik [58] compared projects in three Polish cities: Gdańsk, Poznań, and Lublin. In these cities, the most frequently submitted projects were road ones (footpaths and roadways, driveways, speed bumps, the lighting of pedestrian- and roadways, traffic lights, and road signs). As the research conducted by Bernaciak and Bernaciak indicates [59], in Poznań (532,048 inhabitants in 2020), in the PB 2013–2019, the projects related to human mobility were most commonly aimed at the construction and extension of bicycle paths and the safety of pedestrians. The research findings show an extensive interest of the city dwellers in mobility infrastructure issues.

In many other cities, the dominant type of implemented project was sports and recreational facilities related to spending free time. Such a project was dominant, for example, in Toruń (198,613 inhabitants in 2020), in the years 2014–2017. The most frequently selected projects were sports and recreation infrastructure (including green areas development). In the first three years, road infrastructure projects were ranked as the second, but the share of this type of project declined in the following years. The projects related to “culture and education” were frequently selected. There was an increase in the number of activities addressing “safety, security and order”, e.g., introducing additional lighting, adapting space to people with disabilities, and introducing new litter bins [60].

In Szczecin (398,255 inhabitants in 2020), in the years 2014–2019, among 54 completed projects, the majority referred to recreational areas and greenery (29.6%) and sports facilities (22.2%) [34]. Similar results were recorded for PB in Lublin (338,586 inhabitants in 2020), where, according to the research by Kociuba and Rabczewska [61], in the case of district projects submitted for PB 2017, the largest number of applications was related to leisure and recreation (23.4%), followed by construction or modernization of streets (18.6%) and sports facilities (17.1%). The most popular categories submitted by the inhabitants as part of the city-wide projects were sports facilities (22 projects) and rest and recreation facilities (13 projects).

In some cities, projects related to infrastructure used by children and young people were selected most frequently. For example, in Łódź PB (672,185 inhabitants in 2020), in 2016, the city-wide projects submitted for voting usually concerned “education and upbringing” (36%; usually related to the modernization of schools and their infrastructure), followed by “transport and communication” (27%) [62,63].

The comprehensive comparison of the types of projects selected in the PB 2019 and 2020 in 18 regional capital cities of Poland was performed by Kociuba and Bielecka [41]. In most cities, the highest popularity was assigned to the projects in the field of “rest and recreation” (a total of 1149 projects) and “sport” (456 projects). “Rest and recreation” very often concerned the development of small infrastructure in green areas. Similarly, Mucha [64] carried out the research covering 6061 projects in 243 cities and towns in Poland. The author analyzed three main types of projects: technical infrastructure, social infrastructure, and soft projects, as well as their subtypes. The most common projects were related to sport and recreational functions of urban spaces, to equipping and improving the security of educational establishments and playgrounds for children, and to improving the quality of road infrastructure.

The review of the above articles suggests that, on a nationwide scale, the projects related to human mobility, sport, recreation, and education (e.g., school facilities and areas) were the most frequently implemented tasks in Polish cities. Similarly, in the research by Martel [65], covering 4514 projects from 321 Polish cities, the most frequently implemented ones belonged to such research categories as “space for mobility” and “space for leisure”.

The presented literature shows the preferences of city dwellers expressed through PB; however, it lacks an analysis of the existing correlations between the socio-demographic characteristics of the inhabitants and the projects they choose. The source literature only attempted to assess the relationship between the types of projects and the overall city development. For example, Bernaciak and Kopczyński [66] stated that greenery projects were more frequently submitted in better urbanized regions featuring densely built-up areas. In less developed cities of eastern Poland, inhabitants preferred hard projects raising the level of social and economic development.

In this source literature analysis, the authors deliberately omit the research of rural village funds and urban local grant funds existing in Poland, although in some studies they are presented as one of the forms of participatory budgeting [35,67,68]. There are different procedures for submitting and selecting community projects; therefore, in the authors’ opinion, they should be considered as a different form of increasing social participation in satisfying local needs.

In the following sections of this paper, the authors present the research area and the methodology used in the analysis of the PB in Częstochowa.

## 3. Materials and Methods

### 3.1. Introduction to the Case—The General Characteristics of the PB Rules

Until 2017, there were no separate legal regulations for participatory budgeting in Poland, hence local governments had great freedom in formulating their principles as well as their full voluntary establishment. The PB followed the resolutions passed by the municipal councils or orders issued by the municipal mayors regarding the mode of conducting public consultations [37,45].

Since 2018, PB was formalized through the amendment of the Act on Municipal Self-Government, and basic issues concerning the form of consultations were supplemented [69]. As Article 5a provides: “Within the framework of a civic budget (participatory budgeting), the inhabitants vote directly for their part of municipal budget expenditures each year. The tasks selected in the process are included in the municipal resolution” [69,70]. The Act introduced as obligatory [47,61,69]:

- Establishing PB in cities with “poviat” (county) rights in the amount of at least 0.5% of the municipal expenditure included in the last submitted budget implementation report (there were 66 such cities in Poland in 2021, including Częstochowa);
- Conducting direct voting;
- That the local government specifies the minimum number of signatures required for the submitted application (project) but must not require more signatures under the project proposal than 0.1% of the inhabitants in the area covered by the budget pool in which the project is submitted (i.e., in a district inhabited by 5000 people, a signature of 5 people is enough to submit a project);
- Dividing the funds into independent pools covering the entire municipality and its parts in the intra-municipal units or groups of intra-municipal units (such as urban districts or housing estates).

Most large cities in Poland are divided into intra-municipal units, whose councils, however, have relatively low competences in the area of urban economy planning [71]. Pursuant to the Act, part of the resources at the disposal of PB is divided between such administrative units (the so-called “district projects” or “local baskets”), owing to which their inhabitants have equal opportunities to use “their funds” (for their district), which support the satisfaction of local social needs. The second part of PB funds (usually smaller) is allocated to the city-wide projects (“city-wide tasks”), which may be located throughout the city, concerning more than one housing estate or urban district [47]. This division of PB was also used in Częstochowa.

**Figure 1:**
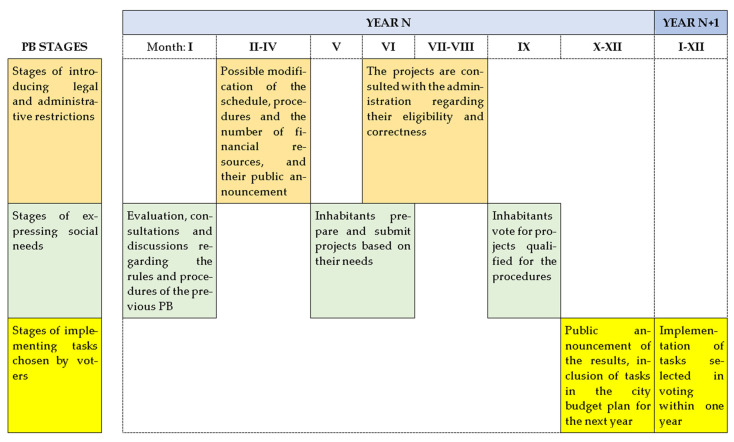
A simplified block diagram of PB procedures in Częstochowa. The breakdown into procedural stages by month is schematic as the dates of the individual stages of the PB change from year to year. Source: Prepared by authors.

A simplified block diagram of cyclical PB procedures in Częstochowa is presented in Figure 1. According to the authors, there are three stages of PB in which inhabitants can express their local needs: the stage of evaluation and consultation regarding the program procedures, followed by the stage of project submission, and finally by the stage of voting on projects. Two stages are related to the introduction of legal and administrative restrictions, i.e., the establishment of the program rules (limitations as to the scope of project works, their location and value) and the stage of qualifying projects for voting by the municipal officials. The projects are prepared by inhabitants and usually submitted in the second quarter of the year (typically May–June). After their formal verification (typically June–July), the lists of projects approved for voting are prepared, and the voting takes place in the third quarter of the year (typically September). As a result of voting, the ranking of projects is determined. Projects are financed for which the funds planned in a given year are sufficient (starting from the first one).

In our research, the focus was on the final stage of project selection, i.e., on the tasks accepted for implementation as a result of voting. We consider them as a reflection of the most important needs expressed by the inhabitants.

### 3.2. The General Characteristics of the Research Area

**Figure 2:**
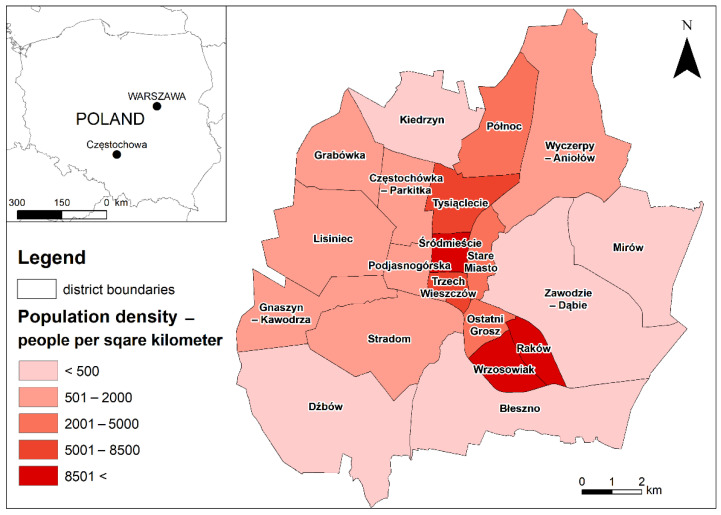
The location of Częstochowa in Poland, the division of the city into districts, and these districts’ population density (people per square kilometer). Source: Prepared by K. Kołat.

Częstochowa is divided into 20 auxiliary city units, called districts, in which their inhabitants can submit and select local projects (Figure 2; Appendix A). The most densely populated districts with the domination of multi-family housing (more than 2001 people per square km) are the central ones located longitudinally along the former (historical) main north–south national road (now transit traffic is directed outside the city via the motorway ring road). Old tenement houses, with relatively high share of people in post-working age (65 and over), prevail in the three central districts (Stare Miasto, Śródmieście, and Podjasnogórska).

Large districts, usually situated in the south and east of the city, are the least populated, and they are dominated by single-family housing (less than 500 people per square km). Single-family housing is developing especially in the north-eastern part of the city. These areas are usually inhabited by people with a higher social status [72]. These districts feature more green spaces and less intense car traffic than the central districts.

According to the 2016 report [73], private cars were the main means of transport for the inhabitants, followed by public transport, whereas bicycle transport was of little importance.

### 3.3. The Subject of Analysis and the Applied Methodology

The analysis covered the data on district projects that met the formal and legal criterion specified by the City Hall in Częstochowa and that were subject to the voting procedure in five editions of PB (years 2015–2019). The analysis uses the division of projects into “hard” and “soft” [74]. The first, hard projects, which were analyzed in the paper, refer to the relatively unique tangible products, such as technical infrastructure, buildings, and plants. They accounted for 78% of all projects submitted in the analyzed PB. The analysis does not include the tasks of equipping libraries, community centers, etc., e.g., the purchase of books and audio storage media. They are not related to the development of public areas. In the case of soft projects, the final result is not a typical tangible asset but the organization of events, promotional campaigns, social education, etc. They were also excluded from this analysis. The other research, which covered 584 cities populated by more than 5000 inhabitants in Poland, also confirmed their inhabitants’ primary interest in hard projects [38,75]. This justifies the possibility of focusing the research on these types of projects, i.e., those that are durable in nature. Ultimately, 1224 submitted projects were qualified for the study of citizens’ activity and project type structure, of which 349 projects chosen by the voting inhabitants were used for the priority needs analysis and correlation analysis.

The authors’ own qualitative typology was applied in their analysis. For this purpose, nine types of projects related to the construction, renovation, or modernization of urban infrastructure or land use were identified. The division used is a qualitative typology based on project description and not a quantitative classification. We do not have numerical data on the detailed scope and value of works in projects. For this reason, we did not analyze the value of the projects. Such a division is acceptable in the methodology of typology [76]. The individual projects could include works, which cannot be analyzed in a completely separate way, related to the different types identified. The projects were assigned to a given type (the acronyms of type used in statistics, tables and figures in the content of the article are given in brackets) based on the main scope of tasks described, according to the following criteria:

- Pedestrian infrastructure (variable acronym: PEDESTRIAN)—separate pedestrian routes (e.g., pavements etc.);
- Bicycle infrastructure (BICYCLE)—separate bicycle paths, installations of bicycle stands, bicycle repair stations, and the infrastructure of city bicycle rental points;
- The road infrastructure (ROAD)—including, e.g., the surface of roads accessible to vehicles and other works within the road lanes, including pedestrian crossings, speed bumps, bus stops and bus shelters, parking lots and parking spaces, traffic lights, road lighting, technical infrastructure running under the roads (including water supply and sanitary sewage systems), and drainage ditches draining the road lane. This category also included tasks that covered the modernization of pedestrian and bicycle routes, together with the road, and cannot be methodically analyzed separately in the PEDESTRIAN and BICYCLE types;
- Sports infrastructure (SPORT)—the construction or modernization of sports facilities not related to educational institutions;
- Educational infrastructure (EDUCATION)—the installation and supplementation of devices, as well as the development and revitalization of the areas within educational institutions; this infrastructure may include school sports facilities;
- Infrastructure and recreational areas (RECREATION)— the arrangement of new areas and the construction of facilities to be used for resting and recreation by the inhabitants, including playgrounds for children, outdoor gyms, and their modernization and supplementation;
- Small architecture (SMALL ARCH)—the installation of small objects supplementing public space, e.g., litter bins, benches, information boards, and lighting;
- Urban greenery (GREENERY)—the development, cleaning, care, and supplementation of green areas, including squares, parks, and separating parks for animals with the installation of devices such as pet waste stations or nesting boxes for birds;
- Other (OTHER)—city monitoring, the comprehensive revitalization of backyards, the installation and renovation of the city sanitary facilities, and the renovation of public buildings.

In order to assess the correlation between the demographic structures of the district inhabitants and their activity in expressing needs within the PB, as well as the structure of project types, the authors used demographic data provided by the Częstochowa City Hall: the number of inhabitants in districts, and the population density and age structure of the population in three occupational categories (in the pre-working age—0–18 years old (children and adolescents), working age—19–64 years old, and post-working age—65 years old or more). At this administrative level, no data are available on the income or education level of the population.

Population density is an indicator that, in the conditions of Częstochowa (and many other large Polish cities), partially reflects the character of the districts. The highest population density is often recorded in central districts dominated by the relatively old multi-family buildings, traditionally inhabited by an aging population presenting either an average or poor financial situation [77,78,79]. In turn, peripheral districts characterized typically by low population density cover the areas with a high share of new single-family housing (terraced or detached), usually inhabited by people with a higher material status. This simplification results from the absence of detailed data on the type of households and the material status of the population. There are no outer slum districts in Poland, such as in some developing countries [80].

The procedures of IBM SPSS Statistics 27 computer program were applied in the analyses of Pearson’s (r) correlation because the analysis covers quotient data. The correlation coefficients were analysed for the three significance levels: *p* < 0.01, 0.01 ≤ *p* < 0.05 [81], and 0.05 ≤ *p* < 0.1 (statistical trend) [82]. In the analysis, predictors mainly take the form of demographic variables (additionally an area of district), while the response variables, containing the data on: (1) voting activity and (2) the type structure of 349 projects (expressed as a percentage—see Appendix A), were selected in voting. The authors realize that a possible correlation does not mean a cause-and-effect relationship, and a potential relation may result from other factors presented in the discussion. The authors also apply traditional methods such as content analysis [83] of municipal reports and strategic documents [73,84,85,86], and a qualitative description [87]. The qualitative data from municipal documents were used by the authors in the general characteristics of the PB procedures and districts, which helped to explain the observed statistical relationships. In the study, the authors consciously do not examine the overall value of projects in districts as the financial resources for districts were allocated in proportion to their population. Thus, such a correlation is determined administratively by the city authorities.

## 4. Results

### 4.1. The Activity of District Inhabitants in Reporting Investment Needs in PB—The Spatial Inventory of Finally Accepted and Rejected Projects

**Figure 3:**
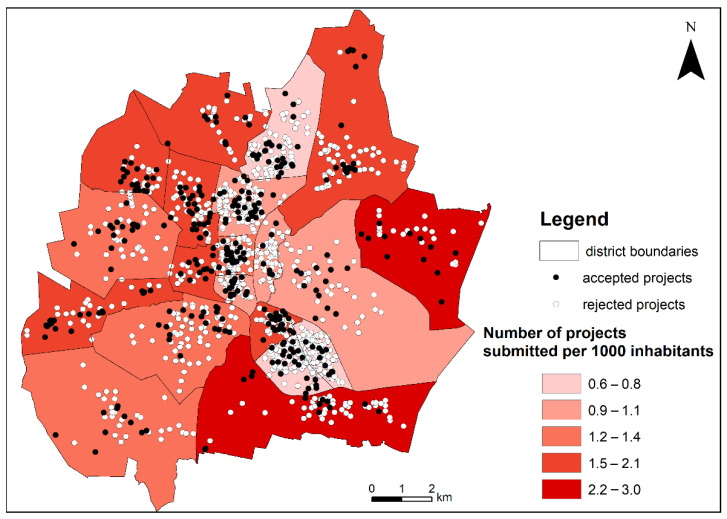
The location of the analyzed district projects submitted in all editions of Częstochowa PB and the indicator of the number of projects submitted per 1000 inhabitants of a given district. Accepted projects—projects that won the vote, accepted to implementation; rejected projects—projects that did not win the vote. Source: Prepared by K. Kołat.

The largest number of projects in the analyzed period was submitted in the densely populated districts such as Tysiąclecie (136 projects), Śródmieście (89), Raków (87), and Wrzosowiak (86) (Figure 3). These districts cover a strip of concentrated multi-family housing with a well-developed social, service, and commercial infrastructure. However, in terms of the number of projects submitted per 1000 inhabitants, these districts were among the least active, especially Wrzosowiak, Raków and Północ districts (indicators from 0.6 to 0.8). These three districts concentrated 33% of all city inhabitants in 2019 [84].

The smallest number of proposals was recorded in peripheral districts with the smallest population: Kiedrzyn (31 projects) and Mirów (31). However, in terms of the relative number submitting the project propositions, these districts were characterized by fairly high activity. The highest average indicator of activity in project submissions by inhabitants over the period of five editions was recorded in Mirów and Błeszno districts, where it reached a value from 2.2 to 3.0 submitted projects per 1000 inhabitants. In 2019, these districts were inhabited by only 3% of the city population [84]. These are outer districts with a high share of new single-family houses inhabited by people of relatively high material status, often working in the nearby industrial districts.

As a result of the voting, most projects were accepted in the relatively densely populated districts such as Tysiąclecie (31 projects), Północ (28), and Częstochówka-Parkitka (27). In relation to the number of submitted projects, most of them were selected in Grabówka (52.8%), Podjasnogórska (46.2%), and Gnaszyn-Kawodrza (44.2%). The lowest values of this indicator were recorded in Raków (11.5%) and Stare Miasto (12.7%)—the districts where multi-family housing is predominant.

Overall, the authors have noted a statistically significant negative correlation coefficient between the population density of the district and the attendance in PB (r = −0.528; Table 1). The districts inhabited by more families with children were more active (a positive correlation coefficient between the attendance in PB and the share of people aged 0–18: r = 0.601). The attendance was also higher in peripheral large area districts (there was a positive correlation coefficient between the area of the district and the attendance in PB: r = 0.763), where there are more green areas and technical infrastructure is less developed. At a low level of significance (0.05 ≤ *p* < 0.1), the statistical trend of lower activity presented by the inhabitants in central districts with a high share of people aged over 65 was also observed.

### 4.2. Typology and Structure of Accepted and Rejected Projects

**Figure 4:**
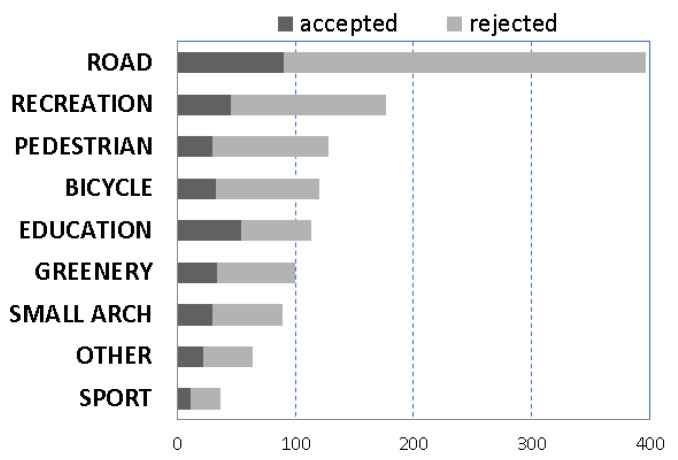
The types of hard projects, accepted or rejected in municipal districts through voting procedures, in Częstochowa PB in the years 2015–2019. Data were sorted by the decreasing number of projects submitted to voting procedures. Accepted projects—projects that won the vote, accepted to implementation; rejected projects—projects that did not win the vote. Acronyms are explained in methodology (Section 3.3). Source: the authors’ compilation based on the data from Częstochowa City Hall.

In the analyzed PB, the inhabitants most often expressed the need for the development of road infrastructure (ROAD type, Figure 4). In this category 394 projects (32%) were submitted, and 90 (23%) of them were voted through for implementation. In the remaining categories, the inhabitants submitted much fewer ideas. The second most frequently submitted group of projects concerned the category of recreation, in which 177 projects (14%) were proposed, and 45 (25%) of them were accepted. The lower numbers of submitted projects covered the following issues, listed in the declining order: pedestrian (PEDESTRIAN) and bicycle (BICYCLE) infrastructure, education (EDUCATION), urban green areas (GREENERY), small architecture (SMALL ARCH), OTHER category, and sports infrastructure (SPORT). Among the projects selected for implementation, their largest number referred to the areas of education (14%) and recreation (11%). In total, 349 projects (29%) out of the 1224 analyzed ones were selected for implementation in the course of voting.

### 4.3. Spatial Distribution of Individual Types of Projects in Relation to the Analysed Features of Districts

**Figure 5:**
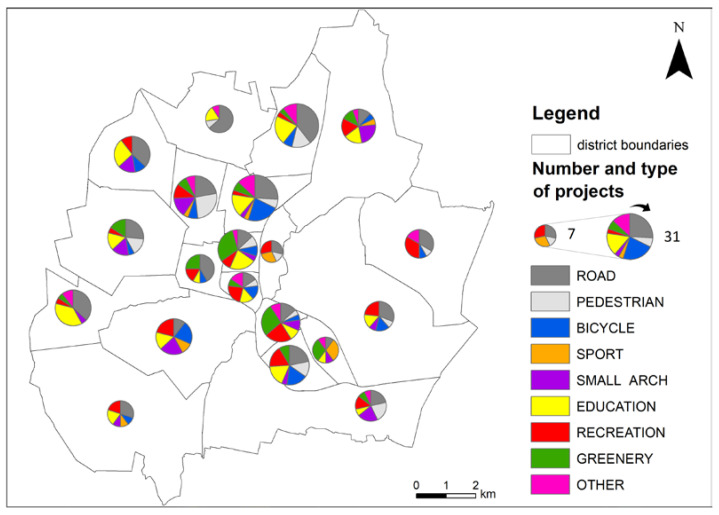
The number and structure of the types of analyzed district projects accepted for implementation in voting procedures in the years 2015–2019. Acronyms are explained in methodology (Section 3.3). Source: Prepared by K. Kołat.

A large diversification of the types of projects selected by the inhabitants for implementation was observed in the majority of the analyzed districts (minimum 4 analyzed types—Figure 5). The road related type of projects (ROAD) was most often implemented in the north-western part of the city (especially Północ and Tysiąclecie districts), but, generally, projects of this type were dispersed in many districts. Pavements (PEDESTRIAN) and bicycle paths (BICYCLE) were frequently chosen in the densely populated districts, along the old north–south communication lane (e.g., Tysiąclecie, Wrzosowiak). Transport traffic accumulates in these districts; therefore, pedestrians and cyclists actively aimed at increasing their safety level. A fairly high concentration of projects was recorded in terms of sports infrastructure (SPORT), which requires relatively large free areas (e.g., various types of pitches, running tracks), hence the possibility of their location is limited. Small scale architecture (SMALL ARCH) and recreation (RECREATION) projects were scattered throughout the city, with their high percentage observed in the moderately populated districts featuring predominantly dispersed or single-family housing (e.g., Stradom and Wyczerpy-Aniołów). The projects addressing educational areas (EDUCATION) were related to schools and kindergartens, which are usually located either inside or in the vicinity of housing estates.

Projects addressing the organization of green areas (GREENERY) were relatively more often selected for implementation in the densely populated districts (e.g., Podjasnogórska and Śródmieście; a statistically significant correlation coefficient between the population density and the percentage share of greenery projects: r = 0.531; Table 2). The exception to this rule was the most densely built-up central Stare Miasto (Old City), where no such projects were selected for implementation. However, it is largely developed and arranged in a way that makes it practically impossible to modernize the terrain function.

In the districts covering a large area, the projects related to small architecture were selected more frequently (a statistically significant positive correlation coefficient between the SMALL ARCH variable and the district area: r = 0.482). These are usually peripheral areas, rich in undeveloped green areas, which were lacking such infrastructure. At the same time, the population number is increasing and the housing areas are being expanded in these sites. Hence, a correlation between the variables “population change” and SMALL ARCH is r = 0.498.

Relatively high (as a percentage) demand for roads extension projects was observed in less populated peripheral districts (a statistically significant negative correlation coefficient between the share of road projects (ROAD) and the district population density: r = −0.464), while for greenery it was observed in densely populated areas (r = 0.531). A negative correlation between the share of the population aged 0–18 and the share of projects addressing bicycle infrastructure (r = −0.472) and greenery (r = −0.485) was observed. Most people in this age category live in peripheral districts with lower building density, less intense car traffic, and more green areas than in the city center. There was also a statistical tendency to select greenery projects in neighborhoods with a high share of people aged 65 and over (r = 0.419). These are central, densely populated districts with dominant old tenement houses and other relatively old multi-family houses.

## 5. Discussion

The analyses carried out within the framework of this article confirm that the demographic characteristics of the population living in particular districts can be related to their activity within PB. However, it is much more difficult to interpret the correlations between age structures and the type of selected projects. This is due to the previously described concentration of the specific population age groups in the districts featuring different types of infrastructure development and land use. Both factors (demographic structures and district development features) have high impact on the preferred project types. It is difficult to separate them in the very general analysis conducted by the authors.

The case of Częstochowa showed that in the densely populated districts with multi-family buildings, the inhabitants’ relative activity in submitting local projects per 1000 inhabitants was lower than in the less populated districts, where single-family housing is more important. It may be related to the higher wealth and education level presented by the inhabitants of single-family estates. Similar characteristics were true for PB in Wrocław [56]. This correlation may also be similar in other cities of Central and Eastern Europe; however, undoubtedly it does not apply to large cities in the countries of the global South, where the outer slum districts are often being developed and inhabited by people with the lowest social and material status. As a result, Polish research findings cannot be universally related to the problems of development characteristics for many other cities in the world [80].

The low activity rate in the submission of projects by the inhabitants of densely populated districts, with predominating old tenement houses and multi-family buildings, may result from their lower social status or the poor social rooting of people who rent this type of housing in the area [78,85]. However, a higher level of infrastructure development in these areas, and thus a smaller number of undeveloped land in relation to the number of inhabitants, may also be significant. In such a case, a smaller number of submitted projects would result from meeting the needs in the field of public infrastructure more successfully and not the effect of low social activity presented by the inhabitants. Additionally, a significant area of the Podjasnogórska central district is protected as a “historical monument”, which makes it difficult to freely locate new infrastructure here [72,88].

People residing in the densely populated central districts of Częstochowa were relatively inactive in voting. These areas are often characterized by a high proportion of people aged 65 and over. Perhaps their low activity is influenced by their exclusion, both digital (fewer people using the Internet in this age group) and physical (high share of people with disabilities) [89]. It may also be influenced by a lower awareness of the possibilities for meeting one’s expectations regarding spatial development in such a formula as PB. Similarly, in Łódź, the voting share of people over 65 years of age was not high [90]. In the majority of Polish cities, the largest number of people vote using Internet forms, which may increase the influence of people actively using this medium [90]. Yet another reason may be the mismatch of needs presented by the oldest age group and the scope of tasks feasible to carry out, for example, older people may have high needs regarding healthcare services and infrastructure. Meanwhile, health services in Poland are provided at the central–national level and do not fall within the competence of a municipality. Since such projects cannot be implemented, senior citizens may not be interested in participating in PB.

The priority of ensuring safety of local mobility complies with the modified concept of Maslow’s hierarchy of needs [91]. During the last dozen or so years, in various cities in Poland, similar results were observed regarding the structure of projects submitted and selected for implementation. This is especially true for the relatively large cities with high population density [56,58,62].

The needs to expand road infrastructure were not concentrated in central districts but were frequently submitted in peripheral areas. This probably results from the fact that the development of road infrastructure did not keep up with the increase in the area of residential building estates and the observed higher number of private motor vehicles [73]. Especially in peripheral districts, with predominant single-family housing, frequent long-term delays occur in the construction of public paved roads, compared to the development of building plots. Separate bicycle paths are not popular in these districts due to the relatively lower level of car traffic. However, this does not result from demographic structures. It is worth noting that bicycle infrastructure in Poland is lagging behind, e.g., compared to the cities in Western and Northern European countries. As a result, projects aimed at separating and developing bicycle paths are very popular among inhabitants [92], especially because as a consequence of higher standard of living, the number of registered cars in Poland has significantly increased in the recent 20 years [93]. Traffic jams and deliberate restrictions on car entry to the city center result in some inhabitants using bicycles, which is reflected in their interest in the development of infrastructure related to this type of transport [94]. Higher social awareness and striving to increase the sense of safety among cyclists and pedestrians in the environment of growing motor vehicle traffic may also be of great importance [95].

High demand for the facilities and areas that meet recreational and sport needs has been recorded in Częstochowa. Projects of this type are often implemented in green areas. This is typical for many PB in Poland [57,60,65] and may indicate the existing insufficient development of this type of public infrastructure. These projects are closely connected with the health of inhabitants who need public places to practice sports or enjoy recreation and relaxation. The underdevelopment of this infrastructure type in cities was probably due to the economic growth-based development concept, dominant in the previous century, which focused on the development of industrial areas, road infrastructure, and commercial services. A higher standard of living resulted in people approaching recreational services related to spending free time as being more important. Increasing the pressure on a healthy lifestyle and the related public infrastructure is a typical tendency in many cities worldwide [53,96]. This largely applies to the development of greenery, the lack of which is experienced by the inhabitants of large cities [97]. In terms of spatial conditions related to the location of projects, the strongest concentration of projects referring to greenery arrangement has been observed in densely populated areas with high share of people aged 65 and older. These are probably the places of their frequent daily resting as they are not a mobile population. In turn, greenery-oriented projects are less popular in peripheral neighborhoods inhabited by an middle-aged immigrants with children (from central districts or outside) as there are relatively many preserved green areas (former forests and farmland).

Greenery projects usually included arranging and equipping these areas with small architecture and small recreational and sports infrastructure (garbage cans, benches, and equipment for practicing sport) rather than increasing their area. In Polish cities, greenery usually constitutes a small percentage of the urban area [98]. Although even undeveloped greenery fulfils numerous important ecological and social functions [97], the pressure from the economic sector and city authorities for their economic or transport use is very strong due to high land prices and a shortage of land that can be used for development and the necessary urban infrastructure [99,100,101]. Additionally, for the city authorities, public green areas usually generate costs (they do not bring budget revenues), while the land sold to a private owner (usually a company or a developer) does generate tax revenues. This causes frequent conflicts between the local communities (often formalizing as non-governmental organizations) attempting to preserve recreational green areas and the city authorities as well as investors striving to develop them [102,103]. The inhabitants clearly express the need, in the form of PB, to preserve green areas and enhance their recreational development.

The structure of projects results from individual ideas and represents the needs expressed by the specific, most active groups of inhabitants or organizations that are able to advertise voting for a particular project [37]. Participation in PB as well as sports and youth education projects is often promoted through schools and sports clubs. This may influence the high participation rate of families with children in voting. PB plays an educational role acting as an incentive enhancing social activity.

It is also worth remembering that the projects, even though resulting from the inhabitants’ needs, may be modified by the limitations of the PB procedures (financial limits and the scope of works possible for funding). As a result, PB can meet only some of the inhabitants’ needs, i.e., those of an exceptionally local nature and a relatively low investment value. This may discourage some people from joining the PB procedures actively, especially in the years to follow, because how many small tasks can be carried out in one’s own small public yard?

A significant weakness of our research is the association of population density with the wealth level of the inhabitants in data interpretation. We believe, however, that in the districts of Polish cities the development of relatively rich peripheral districts is now a frequent phenomenon (because new apartments and single-family houses are relatively expensive) [79]. Additionally, the found correlations are not high, although in social sciences similar values are used to assess the relationship [82]. A great difficulty in research is the lack of quantitative data on education, wealth and the development of individual districts (e.g., the share of single-family houses in the total number of households, land use structure). This type of data would be needed to create a more universal model of the dependence of the needs of inhabitants expressed through the procedures of PB on their socio-demographic structures and the development features of the inhabited districts. In some of our explanations, we relied on qualitative data from city documents, which is acceptable in scientific research [87] but imperfect compared to quantitative methods.

## 6. Conclusions

The collected findings only partially confirmed the adopted hypothesis that the expectations of city inhabitants regarding the development of public spaces may be related to their socio-demographic structures. The conducted research indicates the special importance of families with children (high activity) and people in post-working age (less active). The majority of the projects preferred by the inhabitants can be practically generalized into the two most important types:

- Related to safety and comfort in terms of mobility (construction or modernization of roads, pavements, and separated bicycle paths);
- Related to sports, recreational, and leisure activities in public spaces (including the arrangement of green areas).

The main types of projects are, therefore, related to the issues of the sense of security and physical health (fitness and rest in green areas), i.e., to basic human needs [91]. Demographic structures are closely connected with the processes of district development. In the analyzed city, old, central, multi-family districts with poorer populations became depopulated, leaving a large share of the elderly incumbents—long-term inhabitants attached and used to district development. Greenery projects were chosen more often in these central districts.

In new peripheral districts, where land is cheaper, the development of single-family estates is observed. Such districts are frequently inhabited by the relatively richer working-age people, often with children. Through PB, they try to supplement the underdeveloped infrastructure around the emerging new housing estates, especially in terms of the expansion of roads and landscape architecture. These are usually areas with a greater share of greenery (comparing to central districts) and lower car traffic; therefore, their inhabitants are less likely to choose projects related to bicycle paths and greenery.

In general, the selection of projects is unquestionably determined by both the development state of the district and the demographic structure of the population. These features of districts are often closely interconnected, dynamic, and change slowly within the framework of urban development processes [80].

The authors are of the opinion that participatory budgeting is a valuable way of collecting information about local needs in terms of organizing public spaces. PB voting may be of additional importance as a form of public consultations and guidelines for the city authorities regarding the most urgent needs presented by the inhabitants. The projects that did not win votes but gained relatively high support could be successfully incorporated into other major city investment projects.

PB allows for the identification of local needs for the development or modernization of infrastructure attracting the greatest interest of the local communities. However, because of the selection of projects that received the highest support, the tasks addressing the minorities and the so-called disadvantaged groups (e.g., the disabled) need to receive additional funding as they are less likely to get significant support. The voting method ensures that the projects supported by the most active social groups are actually implemented. Only some of the projects implemented in cities address the architectural facilities related to disabled [55,60]. This social group is not capable of obtaining a high number of votes. Hence, expert investment planning is necessary, which takes into account the facilities indispensable for the disabled, whose needs were previously disregarded in the implementation of public investments in Poland [104]. This indicates that PB should not represent the only form of selecting local investment.

In the future, it would be interesting to carry out research covering the use of infrastructure implemented within the framework of PB. Do the most voted on investments actually have a lot of users? Was the win more the result of both marketing and advertising efforts of a small group of inhabitants or interest groups that do not represent the needs of the majority of inhabitants? Do city governments use voting data as a source of information to prepare strategic development programs? This type of research would also remain the main component in evaluating the undertaken investment activities and would be helpful in the sustainable management of municipal resources.

## Figures and Tables

**Table 1:** The table of correlations between analysed factors and PB voting activity (PB frequency), in Częstochowa districts.

Variable	Coefficients ^1^	Correlation with PB Frequency
Area	r	0.763 ***
	*p*	0.000
Population	r	−0.409 *
	*p*	0.073
Population density	r	−0.528 **
	*p*	0.017
Population change	r	0.426 *
	*p*	0.061
Population aged 0–18	r	0.601 ***
	*p*	0.005
Population aged 19–64	r	0.170
	*p*	0.475
Population aged 65+	r	−0.382 *
	*p*	0.097

^1^ Coefficients: r—Pearson correlation; *p*—statistical significance (two-sided): * 0.05 ≤ *p* < 0.1; ** 0.01 ≤ *p* < 0.05; and *** *p* < 0.01.

**Table 2:** A correlation table of the studied variables characterizing the districts of Częstochowa.

Variable	Coefficients ^1^	ROAD	PEDESTRIAN	BICYCLE	SPORT	SMALL ARCH	EDUCATION	RECREATION	GREENERY	OTHER
Area	r	0.102	−0.057	0.025	−0.155	0.482 **	0.086	0.224	−0.489 **	−0.218
	*p*	0.669	0.813	0.917	0.513	0.031	0.719	0.343	0.029	0.355
Population	r	−0.293	0.121	0.387 *	0.197	−0.187	0.117	−0.380 *	0.169	0.128
	*p*	0.210	0.611	0.092	0.405	0.430	0.624	0.099	0.476	0.592
Population density	r	−0.464 **	0.030	0.182	0.310	−0.359	−0.023	−0.203	0.531 **	0.209
	*p*	0.039	0.899	0.442	0.183	0.120	0.922	0.392	0.016	0.377
Population change	r	0.177	0.047	−0.206	−0.397 *	0.498 **	−0.050	−0.011	−0.129	0.053
	*p*	0.456	0.845	0.383	0.083	0.025	0.835	0.962	0.588	0.824
Population aged 0–18	r	0.276	0.120	−0.472 *	0.070	0.385 *	−0.208	0.254	−0.485 **	−0.062
	*p*	0.239	0.613	0.036	0.770	0.094	0.379	0.280	0.030	0.794
Population aged 19–64	r	0.130	0.279	−0.186	−0.087	0.193	0.037	0.107	−0.281	−0.296
	*p*	0.585	0.233	0.433	0.716	0.415	0.876	0.653	0.229	0.204
Population aged 65+	r	−0.210	−0.261	0.337	0.033	−0.310	0.059	−0.188	0.419 *	0.248
	*p*	0.373	0.265	0.146	0.889	0.183	0.804	0.427	0.066	0.292

^1^ Coefficients: r—Pearson correlation; *p*—statistical significance (two-sided): * 0.05 ≤ *p* < 0.1; and ** *p* < 0.05.

## Data Availability

Archival statistical data available at the Częstochowa City Hall.

## Appendix A

**Table A1:** Basic data on Częstochowa urban districts used in analysis.

No.	District Name	Population 2019 ^1^	Population Change 2007–2019 (%) ^1^	Area sq. km ^1^	Population Density 2019 ^1^	Percentage Share of Population in Age ^2^:			PB Frequency 2015–2019 (%) ^3^	Main Development Feature ^4^
						0–18	19–64	65+		
1	Błeszno	4160	8.0	16.1	259	19.0	65.8	15.2	20.3	scattered development
2	Częstochówka- Parkitka	9112	7.8	4.6	1967	17.0	71.3	11.7	7.2	multi-family blocks
3	Dźbów	5621	−0.1	18.9	297	17.4	67.3	15.3	20.4	scattered development
4	Gnaszyn- Kawodrza	5274	−3.4	7.3	726	16.4	67.1	16.5	13.8	scattered development
5	Grabówka	4419	8.1	6.7	664	17.9	67.7	14.4	7.3	single-family housing estates
6	Kiedrzyn	3047	7.9	8.4	363	19.1	65.3	15.7	17.5	single-family housing estates
7	Lisiniec	9693	6.9	11.1	875	17.2	66.4	16.4	9.7	single-family housing estates
8	Mirów	2301	7.6	12.6	183	19.0	66.1	14.9	20.5	scattered development
9	Ostatni Grosz	8234	16.0	2.0	4170	15.6	64.6	19.9	12.0	multi-family blocks
10	Podjasnogórska	3381	−9.0	2.5	1360	15.1	65.1	19.9	7.1	old tenement houses
11	Północ	27,528	−8.9	6.7	4131	15.5	72.4	12.1	12.9	multi-family blocks
12	Raków	20,747	−13.9	2.1	9824	15.7	65.1	19.2	11.0	multi-family blocks
13	Stare Miasto	10,261	−25.3	2.4	4320	17.6	65.9	16.5	8.8	old tenement houses
14	Stradom	11,794	4.1	11.2	1053	17.0	66.4	16.6	11.5	single-family housing estates
15	Śródmieście	14,250	−16.6	1.6	8818	12.9	63.4	23.7	9.6	old tenement houses
16	Trzech Wieszczów	9649	−18.4	1.3	7249	15.5	64.1	20.4	7.8	multi-family blocks
17	Tysiąclecie	27,081	−13.3	4.3	6308	12.5	56.8	30.7	7.0	multi-family blocks
18	Wrzosowiak	23,908	−19.8	2.8	8551	15.6	70.0	14.4	8.3	multi-family blocks
19	Wyczerpy- Aniołów	8896	−2.2	16.8	530	18.8	68.1	13.1	16.9	scattered development
20	Zawodzie- Dąbie	8673	−5.8	20.3	428	15.6	69.5	14.9	15.2	mixed and industrial areas
	Total	218,029	−9.2	159.5	1377	15.7	66.3	18.0	11.9	

^1^ Our own calculation based on data from Częstochowa City Hall. Population density—population per square kilometer; ^2^ based on [86]; ^3^ the statistical mean of the PB frequency (the number of voters in relation to the district population, in percentage); and ^4^ the typology of the city districts based on [72].
